# Validation of the effectiveness of SARS-CoV-2 vaccines in older adults in “real-world” settings

**DOI:** 10.1186/s12979-021-00248-7

**Published:** 2021-09-22

**Authors:** Nan-ping Weng, Graham Pawelec

**Affiliations:** 1 Laboratory of Molecular Biology and Immunology, National Institute on Aging, NIH, Baltimore, MD USA; 2 Department of Immunology, University of Tübingen, Tübingen, Germany; 3 Health Sciences North Research Institute, Sudbury, ON Canada

**Keywords:** SARS-CoV-2, Vaccination, COVID-19, Longevity of vaccine response, Neutralizing mAb, Aging

## Abstract

The rapidity of SARS-CoV-2 vaccination around the world has substantially reduced the number of new cases of COVID-19 and their severity in highly vaccinated countries. The unanticipated efficacy of SARS-CoV-2 vaccines in older adults has been very encouraging but the longevity of vaccine immunity is currently unknown and protection against emerging variants may be lower. Adoptive immunotherapy with neutralizing mAb may offer an alternative for poor vaccine responders, while the mechanisms underlying failure to respond are still unclear. Further studies of B and T cell responses and their regulation particularly in older populations will provide a more solid foundation to develop suitable approaches to optimize vaccine responses of older adults who fail to mount a durable response.

## Introduction

Vaccination is the most effective way of preventing serious consequences of infections [1]. The alteration of immune function with age has challenged this remarkably effective tool as witnessed by the reduced efficacy of inactivated seasonal influenza vaccines in older adults [2, 3]. Compromised adaptive immunity with aging together with over-active innate immunity is hypothesized to be at least partly due to the lack of sufficient competent antigen-specific B and T cells caused by shrinkage of the immune receptor repertoire of naïve cells, exhaustion of memory cells and dysregulation of innate cells [4]. Despite the initial uncertainty [5], SARS-CoV-2 Spike mRNA and adeno-vectored vaccines in older adults have been surprisingly effective as shown in early clinical trials [6–9] and more recent real-world data in vaccinated old populations [10–13].

Even with the unexpected success of COVID-19 vaccine, some important questions remain to be addressed. The actual longevity of the immune protection induced by vaccination is not yet known, which is especially critical for the old population who are generally less robust immunologically and have lower reserves of resources to deal with challenges. Is an additional vaccine boost necessary for such individuals, and if so when would be the best time? What are the major immune defects of these older adults in terms both of innate immunity and SARS-Cov-2 specific adaptive immunity? What are the available options for these most vulnerable older adults? A better understanding of age-associated changes of immune cells and their regulation is critical for developing specific remedies most needed in older adults.

## Efficacy of COVID-19 vaccines in old populations

The relatively low efficacy of the seasonal influenza vaccine in older populations has been a constant reminder of the need to develop more effective vaccines for older adults whose immune system is considered less robust than their younger counterparts. However, the initial results from the clinical trials of COVID-19 vaccines with a limited number of older recipients (≥ 65 years of age) were unexpectedly encouraging. The Pfizer–BioNTech BNT162b2 mRNA vaccine induced a good antibody titer 7- and 14-days post-second dose in the group of 65- to 85-year-old compared to the younger age group (18–55 years old) [6]. Reports of another mRNA vaccine, the Moderna mRNA-1273 in 40 older adults (56 to 70 years or ≥ 71 years) showed that both binding- and neutralizing-antibody responses were similar to those in the younger age group (18–55 years old) and were higher than the median titer of convalescent serum [7]. In addition, a strong CD4 cytokine response involving type 1 helper T cells post-vaccination was also observed. The Astra-Zeneca ChAdOx1-S vaccine also induced good anti-spike SARS-CoV-2 IgG and neutralizing antibody titers 28 days after the second dose in the over-70’s [8]. In addition, T-cell responses were detected at day 14 after a single dose of the ChAdOx1 vaccine. However, the significance of these promising results based on the limited number of healthy old vaccine participants requires real-world evaluation.

A recent series of publications that examined the efficacy of these COVID-19 vaccines in the real-world has confirmed these findings from the clinical trials and provided new information. First, analysis of the effectiveness of the BNT162b2 and ChAdOx1-S vaccines for preventing confirmed Covid-19 symptoms, admissions to hospital, and deaths of 156,930 adults aged 70 years and older in the UK showed that the effectiveness of BNT162b2 is 61% (51 to 69%) from 28 to 34 days after vaccination whereas the effectiveness of ChAdOx1-S is 73% (27 to 90%) from day 35 onwards [10]. A single dose of either vaccine has ~ 80% effectiveness at preventing admission to hospital with COVID-19 and a single dose of BNT162b2 has 85% effectiveness at preventing death with COVID-19. An even better result from Israel shows that the effectiveness of two doses of BNT162b2 is 94.8% in the 65-year-old or over group [11]. It must be borne in mind that all these data derive from studies of vaccinating recipients with ancestral virus Spike antigens and assessing protection against the same virus, prior to the emergence of numerous variants. This is a very important proviso, because ancestral-form vaccines do not protect anywhere near as well against, e.g., the Delta variant.

The general state of health of older adults is highly variable, and participants in clinical trials are likely healthier than those in the general older population. In this respect, the residents of the long-term care facilities (LTCF) are likely reflecting the less healthy older adults. Two recent reports analyzed BNT162b2 vaccine effectiveness in LTCF residents: one examined 39,040 LTCF residents (median age at first dose; 84 years, age range: 77–90) in Denmark and found that there was no protective effect after the first dose but 64% (95% CI; 14–84) after the second [12]. Another report examined LTCF residents in Spain and found 71% (95% CI: 56–82%), 88% (95% CI: 75–95%), and 97% (95% CI: 92–99%) effectiveness against SARS-CoV-2 infections (symptomatic and asymptomatic), COVID-19 hospitalizations and deaths, respectively [13]. These findings illustrate substantial differences in COVID-19 vaccine effectiveness between the healthy and less healthy older populations. Approximately one-third of those less healthy older populations did not have a protective vaccine response and were still vulnerable to the SARS-CoV-2 infection. Thus, there is an urgent need to develop strategies to help this group of older adults.

## Strategy for treating poor vaccine responders

The ultimate solution for treating those older poor vaccine responders relies on a better understanding of the mechanisms underlying the general age-associated dysregulation of immunity as well as any individual-specific defects. The longer-term goal is to design vaccine and immunization schemes specifically for the older populations including increased dose, different adjuvants, and heterologous vaccination. At the moment, passive immunization or serotherapy remains an available option which has been used to combat infectious disease for over a century [14]. Over the past year, numerous monoclonal antibodies have been developed against SARS-CoV-2 proteins. To date, six mAbs have received an Emergency Use Authorization (EUA) from the FDA and several additional mAb are being evaluated in phase 3 clinical trials. The application of these mAbs covers the whole gamut from prophylaxis to therapy for early- or late-COVID-19 patients [15]. These neutralizing mAbs are targeted to the receptor-binding domain (RBD) of the S glycoprotein of SARS-CoV-2 and block its binding to the ACE2 protein to prevent the entry of the virus into target cells [16]. This is considered the main mechanism of immune protection by neutralizing mAbs (i.e., “sterilizing” immunity). However, the role of the Fc effector functions of neutralizing mAbs has not been fully characterized and it remains to be determined whether this pathway is used to facilitate viral clearance on the one hand, or whether it enhances undesirable effects such as inflammation on the other.

The initial clinical trials of the neutralizing mAb (LY-CoV555) were targeted to mild-to-moderate COVID-19 in adults and pediatric patients 12 years and older and showed a promising ability to accelerate the natural decline in viral load over time [17]. A cocktail of neutralizing mAb (REGN-COV2) used for treating mild-to-moderate COVID-19 in adults at high risk of progressing to severe COVID19 also effected a reduced viral load in patients who were serum SARS-CoV-2 antibody-negative [18]. However, the effect of neutralizing mAb therapy in hospitalized COVID-19 patients was not as effective as with mild-to-moderate COVID-19 disease. Although there are no published results of prophylactic application in the old population, two ongoing clinical trials show promising interim results [15] and thus give hope that neutralizing mAb can be an effective alternative to COVID-19 vaccination for those older adults who have a poor vaccine response. The remaining practical questions are how long the passive infusion of neutralizing mAb will be effective in vivo and whether an additional dose is required for an effective protection over a longer time, such as 6 months or 1 year.

## Longevity of vaccine immunity against the ancestral and variant strains

The success of vaccination in reducing new COVID-19 cases and severe disease is assessed weeks or months post-vaccination. Studies of the adaptive immune response of COVID patients show antigen-specific B and T (both CD4 and CD8) cell responses [19] and this induced immune protection is effective for up to 1 year [20, 21]. But how much longer vaccine-induced immunity remains effective against SARS-CoV-2 infection cannot yet be known. Considering age-related differences in immune system functionality, it will be interesting to determine whether vaccine immunity is shorter-lived in older than younger adults. Preliminary data certainly suggest that this is likely to be the case (see https://www.ndm.ox.ac.uk/files/coronavirus/covid-19-infection-survey/finalfinalcombinedve20210816.pdf). Like the measure of influenza vaccine response, the neutralizing antibody titer of COVID-19 vaccine is considered as a major indicator of immune protection from infection and disease. In the case of mild COVID-19 patients, a three-month follow-up study showed that antibodies capable of neutralizing SARS-CoV-2 were present in most people [22]. Another study showed that neutralizing antibodies can be detected up to 10 months after symptom onset [23] but it is unclear whether vaccine-induced neutralizing antibody titers lasts for a similar length of time. Determination of an optimal time for administering a vaccine booster is critically important for both amplifying immune protection and for practical feasibility. Finally, emerging variants of SARS-CoV-2 need to be taken into consideration in the design of the booster vaccine. Neutralizing antibody longevity and cross-reactivity to different variants are equally critical for the success of vaccination. The initial result of the above study shows that neutralization of the Beta variant (B.1.351) is lower compared to the original strain [23]. Thus, a vaccine booster provides an opportunity for using a modified vaccine design to improve effectiveness of neutralizing antibodies against the variants.

Cell-mediated immunity plays an essential role in the control of viral replication and eventual viral clearance. Studies of COVID-19 patients have shown a rapid T cell response to different SARS-CoV-2 proteins including S protein [19, 24] and the clinical trials of COVID-19 vaccines have also documented T cell activation [7, 8]. More importantly, viral epitopes recognized by T cells are often highly conserved and T cell recognition of peptide/MHC complexes are relatively degenerate, encouraging cross-reactivity. Indeed, a study of T cell response before and after the mRNA vaccination showed that spike protein-specific T cells respond similarly to the epitopes from the ancestral (B.1.1.7) and the Beta variant strain (B.1.351) [25]. This suggests that cell-mediated immunity may offer a broader protection than neutralizing antibodies against viral variants. Although the duration of cell-mediated immunity to COVID-19 vaccine also remains to be determined, studies of SARS-CoV infection showed that T cell-mediated immunity offers a longer protection than neutralizing antibodies [26]. If this turns out to be the case for COVID-19 vaccine-induced immunity as well, we might be able to delay the boost dose based on T cell responses.

## Concluding remarks

COVID-19 vaccination has yielded encouraging results in older populations in the real world following clinical trials, but there may still be a need for supplementary treatments for the older population in general and for others suboptimal responders. Serotherapy is currently an alternative for those older adults that respond poorly to vaccination. The longevity of vaccine immunity and its effectiveness against different variants are uncertain and will need more carefully designed longitudinal studies. The immune response to SARS-CoV-2 is a highly coordinated process and various level of dysregulation could contribute to the failure of effectively combating the disease in older adults [19]. Thus, a better understanding of the mechanisms underlying the age-associated changes of the immune system will lead to further improvement of vaccine efficacy for SARS-CoV-2 variants and development of additional therapeutic options for the poor older vaccine responders.
